# Identification and analysis of gene families from the duplicated genome of soybean using EST sequences

**DOI:** 10.1186/1471-2164-7-204

**Published:** 2006-08-09

**Authors:** Rex T Nelson, Randy Shoemaker

**Affiliations:** 1 USDA-ARS CICGR, Iowa State University, Ames, IA, 50011, USA

## Abstract

**Background:**

Large scale gene analysis of most organisms is hampered by incomplete genomic sequences. In many organisms, such as soybean, the best source of sequence information is the existence of expressed sequence tag (EST) libraries. Soybean has a large (1115 Mbp) genome that has yet to be fully sequenced. However it does have the 6th largest EST collection comprised of ESTs from a variety of soybean genotypes. Many EST libraries were constructed from RNA extracted from various genetic backgrounds, thus gene identification from these sources is complicated by the existence of both gene and allele sequence differences. We used the ESTminer suite of programs to identify potential soybean gene transcripts from a single genetic background allowing us to observe functional classifications between gene families as well as structural differences between genes and gene paralogs within families. The identification of potential gene sequences (pHaps) from soybean allows us to begin to get a picture of the genomic history of the organism as well as begin to observe the evolutionary fates of gene copies in this highly duplicated genome.

**Results:**

We identified approximately 45,000 potential gene sequences (pHaps) from EST sequences of Williams/Williams82, an inbred genotype of soybean (*Glycine max *L. Merr.) using a redundancy criterion to identify reproducible sequence differences between related genes within gene families. Analysis of these sequences revealed single base substitutions and single base indels are the most frequently observed form of sequence variation between genes within families in the dataset. Genomic sequencing of selected loci indicate that intron-like intervening sequences are numerous and are approximately 220 bp in length. Functional annotation of gene sequences indicate functional classifications are not randomly distributed among gene families containing few or many genes.

**Conclusion:**

The predominance of single nucleotide insertion/deletions and substitution events between genes within families (individual genes and gene paralogs) is consistent with a model of gene amplification followed by single base random mutational events expected under the classical model of duplicated gene evolution. Molecular functions of small and large gene families appear to be non-randomly distributed possibly indicating a difference in retention of duplicates or local expansion.

## Background

When available, the information provided by whole-genome sequencing projects provides an entry into an understanding of genome structure and evolution and gene discovery, and function. Unfortunately, the size and complexity of the genomes of many agronomically important species currently hinders the undertaking of such projects. Due to its economic importance, a publicly funded whole-genome sequencing effort will soon be undertaken, however the size and complexity of the soybean genome (1115 Mbp; [1]) may delay a full sequence assembly. In the interim, the majority of efforts at gene discovery for many organisms, including soybean, has been through the sampling and partial sequencing of gene transcripts (expressed sequence tags or ESTs) [2]. Such EST data form a valuable foundation for the understanding of the gene composition and genomic biology of yet-to-be fully sequenced genomes [3].

Band-counting using RFLP probes indicates that more than 90% of all low copy sequences in soybean are present in more than two copies [4]. Consistent with this, detailed genetic mapping using hybridization-based RFLP markers and multiple populations identified many instances of duplicated genomic regions [4]. The presence of "nested" duplications suggested that at least one of the original genomes had been duplicated prior to the most recent polyploidization event [4,5]. Thus it is expected that most soybean genes occur in gene families consisting of two or more paralogs. In a recent study, Schlueter *et al*. [6] analyzed ESTs from duplicated genes and concluded that the soybean genome underwent major duplication events at approximately 15 and 44 MYA. The more recent duplication event in particular would be expected to result in many paralogous pairs of genes differing by relatively few sequence differences, thus complicating gene identification using ESTs. Although some preliminary studies have examined the level of sequence variation between selected genes and their alleles in soybean [7], no systematic analysis of this important subject has been done until now.

Analysis of EST libraries has been proposed as a way to identify most of an organism's genes and gene families, as well as their alleles in other genotypes. Unfortunately, since ESTs are single-pass reads, the sequence error rate may often approach or exceed the sequence differences between paralogs or alleles [7-9]. Additionally, because the ESTs are usually derived from multiple genetic backgrounds, it is often difficult to partition observed sequence differences between ESTs to paralogs and their alleles [3,10]. Despite these recognized problems, ESTs have been used in several plant and animal species to identify genes expressed in tissues or whole organisms [11-17]. The main advantages to using ESTs to identify genes are that they are easily produced and, since they represent coding sequences, they directly identify the gene of interest.

We have developed a series of informatics steps which minimize the problems inherent in using ESTs for gene identification [18]. First, by constraining our analysis to a single homozygous genotype we markedly reduce any ambiguity in distinguishing members of a multigene family from alleles of a single gene. Second, combining Cap3 EST clustering and BLAST analysis, we were able to utilize their unique strengths to identify genes and, when appropriate, assign them to gene families and thirdly, by employing a redundancy criterion, we identify sequence differences between the closely related genes in gene families which occur more than once, thus reducing the chances of accepting sequencing errors in the dataset. Once the genes that constitute those families have been identified, observations on the distribution of sequence differences provide a glimpse of gene and paralog evolution within the gene families.

## Results

### Contig assembly

The ESTminer suite of programs [18] uses as input output files from both Cap3 and BLAST. Cap3 [19] is used to initially construct contigs which represent the consensus sequence of families of highly related genes. The default settings were used with the exception of -o 21 and -y 10. The Cap3 parameters were adjusted with the goal of including all members of a gene family in the Cap3 consensus sequence, even though this also allowed the inclusion of some ESTs that were only distantly related or whose shared similarity was based on only a relatively short motif. Clustering of the 196,867 All-Williams (AW) ESTs using Cap3 resulted in 17,463 Cap3 consensus sequences (gene family consensus sequences) and 56,430 Cap3 singletons. Preliminary analysis of these results revealed that Cap3 did not consistently include all of the related ESTs in an alignment. To overcome this limitation, ESTminer requires the use of each Cap3 consensus sequence as the query in a BLAST [20] search of the entire AW EST collection. This step allows all EST sequences the opportunity to be included in a family assembly based on primary nucleic acid sequence. An additional quality control step is used to remove EST sequences from a family assembly that were included by BLAST based only on short "motif" similarities by requiring each BLAST hit length to be 90% of the length of the EST sequence. Finally a redundancy criterion is applied to each variable position in the family alignment before a sequence is validated as a separate gene sequence thus reducing the inclusion of sequences differing by random cloning or sequencing errors as separate gene sequences.

### Cap3 singleton analysis

The family consensus sequence creation step carried out by Cap3 also produced 56,430 unique Cap3 singletons representing EST sequences which did not have a significant match or overlap in the dataset to be included in a Cap3 family consensus sequence. The ESTminer program allows all of the AW ESTs to participate in defining potential gene sequences (pHaps), including those initially identified by Cap3 as singletons. Interestingly, 6,535 (11.6%) of the Cap3 singletons were aligned by BLAST and were used by ESTminer to identify pHaps. The remaining 49,895 (88.4%) Cap3 singletons were subjected to BLASTX analysis of proteins from the viridiplantae contained in GenBank at an expectation cut-off of 1 × 10^-4^. Twenty- five percent (14,109) had no significant hit to the database indicating that they represent either rarely expressed soybean specific genes or are technical artifacts of the EST cloning process. Thus 35,786 (63.4%) of the Cap3 singletons presumably represent soybean homologs to known plant genes which were poorly represented in the tissues and conditions from which the EST libraries were prepared.

### Gene family analysis

Cap3 assembly produced 17,463 gene family consensus sequences. These sequences along with the BLAST output file were processed using the ESTminer programs[18]. After validation 12,702 families could be further analyzed. Table 1 presents the distribution of the number of pHaps (potential gene sequences) in these gene families. The largest class of families contain a single pHap (67%) indicating that all of the included EST sequences were identical in their overlap region and are therefore considered one sequence. Families with 2 to 10 pHaps constitute 28% of all the families, while families with more than 10 or more pHaps constitute only 5% of the families. Thus 95% of all gene families appear to have less than 10 members. The average number of pHaps in a family was calculated to be 9.

**Table 1:** Distribution of pHaps Among 12,702 Gene-families

Potential Haplotypes per Gene family (45,255 total pHaps)	Frequency in the Gene families (% of total)
1	8,535 (67%)
2	1316 (10%)
3	706 (6%)
4	472 (4%)
5	320 (3%)
6	205 (2%)
7	190 (2%)
8	134 (1%)
9	89 (< 1%)
10	85 (< 1%)
>10	651 (5%)

### Gene analysis

**Figure 1:**
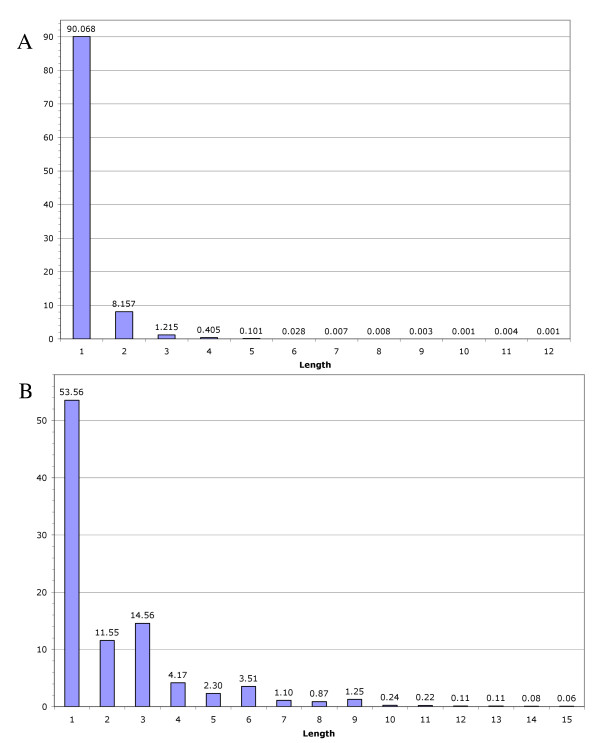
**Distribution of insertion lengths and consecutive substitutions within gene families**. A) Consecutive base substitutions demonstrate that single base substitutions are the primary size class, consisting of 90% of all substitutions which reduce in number rapidly. The largest consecutive stretch of substitutions was 12. B) Insertion lengths in terms of percent of the total number of insertion events are shown. Insertion lengths demonstrate excess insertions of lengths 3, 6, and 9 bases however, the largest size class is single base Insertions which compose 58% of all insertion events. The data shown is for insertions less than 16 bases in length.

Potential gene and paralog sequences (pHaps) within a gene family are differentiated by one or more sequence differences termed locus defining sequence differences (LDSDs). As an initial step in understanding the mechanisms by which genes within a gene family diverged, we analyzed the gene sequences for the types and numbers of LDSDs they contained. Analysis of the 12,702 gene families identified 45,255 pHaps. Detailed examination of the types of sequence differences which define separate pHaps within a family allowed the identification of 10,683 short insertion and 114,301 substitution events in the dataset. Non-consecutive single base substitutions were the predominant form of sequence variation accounting for 90% of all base substitution differences (Figure 1A). Interestingly, two and three consecutive base substitutions accounted for 8% and 1%, respectively, of all substitution events. Although relatively rare, longer runs were observed with the largest seen being 12 consecutive base substitutions. The frequency of base substitution LDSDs for the dataset as a whole was 15.8/kb (Table 2). Since 67% of all gene families contained only a single potential gene sequence (pHap) and thus displayed no sequence variation, we reasoned that their presence in the calculation biased the frequency estimate. If only gene familes with more than one pHap are considered in the calculation, the base substitution frequency more than doubles to 40.3/kb.

**Table 2:** Base Substitution and Indel Frequencies within Gene families

Type of LDSDS	Transcriptome Wide	Restricted to Families with >1 pHap
Base Substitutions	15.8/kb	40.3/kb
In/Dels	1.3/kb	3.4/kb

Examination of insertion LDSDs showed that 1 bp insertions are the predominant size class (Figure 1B). This result was unexpected as a single or even paired single base insertions would be expected to disrupt the reading frame. Although there were insertions found up to 1.6 kb, there were few with lengths greater than 15 bases. As expected, insertions of three or multiple of three bases are more frequent than would be predicted from the baseline, suggesting that they were subject to less selection pressure. The frequency of insertion LDSDs in the whole dataset, 1.3/kb, was less than that of base substitutions LDSDs (Table 2). When restricted to families with more than one pHap the insertion frequency tripled to 3.4/kb. The average number of insertions in these families was 2.6 and the average size of each insertion was 7.8 bp.

Because 90% or more of the "single" member families may have more than one member in the genome (see below), most "single" member families typically represent gene families with few members. However, the transcriptional competence of those sequences are not known since they were not represented in the EST collection. Therefore, we estimate that the rate of substitution LDSDs defining genes within families is between 15.8 – 40.3/kb and the insertion LDSD rate is 1.3 – 3.4/kb. Thus, on average, two different pHaps (genes in the same ESTminer gene family) that are 1 kb in length will differ by 16 – 40 base substitutions and 1 – 3 insertions.

### LDSD validation study

In the context of these experiments, LDSDs represent the sequence differences which define genes in an ESTminer gene family. They differ from single nucleotide polymorphisms (SNPs) in that SNPs define differences between alleles of a gene in different genetic backgrounds whereas LDSDs are the sequence differences between different genes of a gene family in the same genetic background. To attempt to determine the validity of the LDSDs identified by ESTminer, a small sampling of single and multiple pHap families was conducted. Nine examples of single and 4 multiple gene families were randomly chosen for partial genomic DNA amplification and sequencing. PCR amplification of 8 (89%) of the 9 single-pHap families with primers designed from their single representative pHap produced two or more bands (data not shown). Examination of the sequence traces from the most prominent band of each amplification indicated that single base insertion/deletions made up the majority of all sequence discrepancies between the pHap sequence used to design the PCR primers and the genomic locus sequenced. As expected, all of the multiple-pHap family amplifications produced multiple PCR products (data not shown) indicating that there is variation in gene structure between the highly similar genes within a family. A total of 4,597 bp of coding sequence were covered by pHap sequence in the 13 loci examined. Thirteen large insertions were encountered in the genomic sequence of the 13 loci compared to their EST derived pHap sequence. The average size of insertion was 220 bp and all insertions completely sequenced conformed to the 5' – GT...AG – 3' intron splice site consensus and are therefore presumptive introns.

A total of 83 non-indel gene defining polymorphic sequence positions (LDSDs) were included in the regions sequenced. At three (4%) LDSD positions, the base present in the genomic sequence examined was not one of the bases observed in the EST collection. Since the pHap sequences were based on EST sequences, it cannot be determined if these represent LDSDs that define genes which were not represented in the libraries, are non-expressed pseudogenes or are some type of PCR error introduced during the amplification step prior to sequencing. But assuming they are errors it indicates that, on the whole, the LDSDs are approximately 96% accurate, thus the non-indel predicted LDSDs appear to be highly reliable in terms of both base prediction as well as indicating variable positions in each sequence.

### Functional prediction of gene families and potential gene sequences (pHaps)

**Figure 2:**
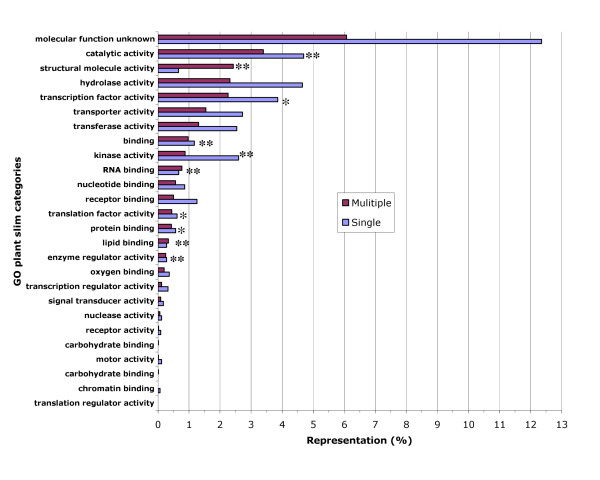
**Distribution of GOslim terms among gene families**. Histogram of GOslim terms associated with all gene families. Red bars indicate gene families with multiple genes and blue bars represent gene families which were composed of a single gene. A single asterisk indicates a significant departure from independence in a Chi-square test (1df, p ≤ 0.05) and a double asterisk indicates a probability level of p ≤ 0.01. In general, families composed of few genes (single) made up the majority of all family types in each category with the exception of the categories of structural molecule activity, RNA and lipid binding where multiple gene families appear to be in the majority.

To assign functional annotations to the gene family and pHaps we compared the family and pHap nucleic acid sequences to the highly annotated protein sequences contained in the Gene Ontology consortium product SeqDBlite [21] by BLASTX analysis. A series of Perl programs were used to parse the BLASTX output and tabulate the results (Table 3 and 4). Figure 2 presents the functional annotations associated with the gene families using the Plant GOslim terms for clarity.

**Table 3:** Distribution of GOslim terms among ESTminer gene families

GOslim Category	Multiple	Single
Chromatin Binding	0	8
carbohydrate binding	2	0
motor activity	2	15
receptor activity	3	11
nuclease activity	7	15
signal transducer activity	11	32
transcription regulator activity	15	41
oxygen binding	25	46
enzyme regulator activity	31	298
lipid binding	42	35
protein binding	55	73
translation factor activity	56	78
translation regulator activity	0	1
receptor binding	63	160
nucleotide binding	72	109
RNA binding	97	85
kinase activity	111	330
binding	123	149
transferase activity	166	323
transporter activity	196	346
transcription factor activity	287	490
hydrolase activity	294	591
structural molecule activity	308	84
catalytic activity	431	596
molecular function unknown	771	1569
No Functional Annotation	999	3050

**Table 4:** Distribution of GOslim terms among individual ESTminer potential gene sequences (pHaps).

GOslim Category	Multiple	Single
Chromatin Binding	0	10
translation regulator	0	1
motor activity	2	14
carbohydrate binding	5	0
receptor activity	10	11
nuclease activity	27	12
signal transducer	32	16
transcription regulator	40	40
oxygen binding	100	47
enzyme regulator	298	36
kinase activity	402	297
lipid binding	407	34
translation factor	431	78
nucleotide binding	525	104
RNA binding	659	83
protein binding	898	84
transferase activity	1160	335
transcription factor	1311	441
binding	1408	156
transporter	1460	334
hydrolase activity	1950	558
structural molecule	2982	83
catalytic activity	3569	634
receptor binding	3725	165
function unknown	4861	1573
No Functional Annotation	10458	3389

GO terms form a tree-like network with specific annotations at the termini or leaves and increasingly general terms as branches which eventually lead the most general terms for biological functions near the origin of the ontology or "root". GO slim terms are arbitrarily chosen from internal branch nodes close to the "root" of the ontology to represent all of the more granular "leaf" terms associated with it. The effect of this is to allow the observer to more easily see which terms are related to the larger biological processes without intimate knowledge of all biological functions, processes and the cytological compartments where these functions or processes take place.

**Figure 3:**
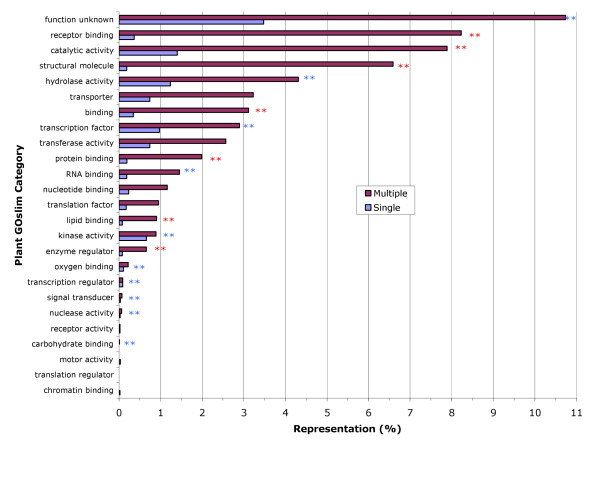
**Distribution of GOslim terms among individual genes**. Histogram ofGOslim terms associated with all genes. Red bars indicate genes from multiple gene families (multiple) and blue bars represent genes from families with few members (single). Asterisks indicate comparisons where multiple gene families contained more (Red) or fewer (Blue) members than expected. Significance was judged at the 0.05 probability level (single asterisk) using a Chi-square test in each category. Double asterisks indicates significance at the 0.01 probability level. Genes from multiple member families are the predominant class of genes in each category. The pHaps were not randomly distributed among the GO categories with proteins involved in kinase, hydrolase, oxygen binding, transcription regulator, nuclease, signal transducer and transcription factor activities appearing to contain fewer members than the average multiple gene family while families in the categories of enzyme regulator structural molecule and catalytic activity and receptor, protein and lipid binding appear to have larger than average multiple gene families.

The bars in Fig. 2 represent the percent of the total number of families that are associated with each category. A chi-square test was performed on the raw data for each GO category. This analysis indicates that there are significant differences in the distribution of GO slim categories among the gene families with some Chi square values exceeding critical values by 10 fold or more. Since 67% of all ESTminer gene families contained few members (single), gene familes with few members were the predominant type in most GO categories. The exceptions were RNA and lipid binding and structural molecule categories. In these categories there were more gene families with many genes (multiple) than gene families with few members (single). Not surprisingly, a similar comparison of the percentages of total pHaps in a GO category in terms of each type (single, multiple) of gene family indicated that most pHap sequences in each category were from families with many sequences (Figure 3).

In order to determine if any of the GOslim categories contained multiple gene families composed of an unusually large or small number of genes, we examined the number of individual genes (pHaps) within each multiple gene family in each GOslim category (Figure 3). In order to provide an expected value we calculated the average number of members in a multiple gene family to be 9. Since single gene families contain, by definition, a single potential gene sequence (pHap) the number of single sequence gene families equals the number of pHaps from single gene families. Thus, for a given GOslim category, if the numbers of families from single pHap families equals the number of multiple gene families, then there should be 9 times the number of pHaps from multiple gene families than are in single member families. Analysis of these data indicate that the size of the multiple gene families vary in various GO categories with 7 categories containing multi-gene families larger than the average (Fig. 3 red asterisks) and 10 categories containing families with fewer than average number of members (Fig. 3 blue asterisks). Again, Chi-square values exceeded critical values by many fold indicating significant departures from expectations.

Comparing Figure 2 to Figure 3 for the categories of RNA and lipid binding and structural molecule activity, while these categories had more multiple gene families than expected, the size of the individual families vary suggesting that there is no common mechanism for this observation. From Figure 3, it appears that the size of the multiple gene families for lipid binding and structural molecule activity are larger than expected however, the size of the multiple gene families in the category of RNA binding is smaller than expected.

Further analysis of the gene family GO functional annotations was carried out using the program Blast2Go [22]. This program calculates the Fisher's Exact Test and determines the FDR (false discovery rate) as well as identifies individual GO terms which were significantly enriched in a test set of sequences compared to a reference set. This analysis identified 50 GO terms which were significantly over-represented in the single pHap gene families compared to the entire gene family dataset (Table 5). The transferase categories made up the largest portion of the terms (36%) with permeases/transporters the next most abundant class (18%).

**Table 5:** Enriched GO terms in gene families with few members (single)

**GO ID**	**GO Term**
GO:0016740	transferase activity
GO:0016772	transferase activity, transferring phosphorus-containing groups
GO:0008170	N-methyltransferase activity
GO:0008276	protein methyltransferase activity
GO:0042054	histone methyltransferase activity
GO:0016757	transferase activity, transferring glycosyl groups
GO:0016773	phosphotransferase activity, alcohol group as acceptor
GO:0016758	transferase activity, transferring hexosyl groups
GO:0016279	protein-lysine N-methyltransferase activity
GO:0018024	histone-lysine N-methyltransferase activity
GO:0046974	histone lysine N-methyltransferase activity (H3-K9 specific)
GO:0046976	histone lysine N-methyltransferase activity (H3-K27 specific)
GO:0016278	lysine N-methyltransferase activity
GO:0005554	molecular function unknown
GO:0008757	S-adenosylmethionine-dependent methyltransferase activity
GO:0008194	UDP-glycosyltransferase activity
GO:0016410	N-acyltransferase activity
GO:0016407	acetyltransferase activity
GO:0008080	N-acetyltransferase activity
GO:0003682	chromatin binding
GO:0030554	adenyl nucleotide binding
GO:0005524	ATP binding
GO:0003700	transcription factor activity
GO:0030515	snoRNA binding
GO:0030599	pectinesterase activity
GO:0016301	kinase activity
GO:0004672	protein kinase activity
GO:0004674	protein serine/threonine kinase activity
GO:0016538	cyclin-dependent protein kinase regulator activity
GO:0004428	inositol or phosphatidylinositol kinase activity
GO:0015291	porter activity
GO:0015290	electrochemical potential-driven transporter activity
GO:0015171	amino acid transporter activity
GO:0005275	amine transporter activity
GO:0015203	polyamine transporter activity
GO:0005279	amino acid-polyamine transporter activity
GO:0015359	amino acid permease activity
GO:0005342	organic acid transporter activity
GO:0046943	carboxylic acid transporter activity
GO:0016789	carboxylic ester hydrolase activity
GO:0016788	hydrolase activity, acting on ester bonds
GO:0016810	hydrolase activity, acting on carbon-nitrogen (but not peptide) bonds
GO:0016811	hydrolase activity, acting on carbon-nitrogen (but not peptide) bonds, in linear amides
GO:0004722	protein serine/threonine phosphatase activity
GO:0004721	phosphoprotein phosphatase activity
GO:0008026	ATP-dependent helicase activity
GO:0004386	helicase activity
GO:0008236	serine-type peptidase activity
GO:0004803	transposase activity
GO:0003777	microtubule motor activity

## Discussion

We employed ESTminer to analyze an EST collection to identify the genes represented in the collection. ESTminer identifies potential haplotype sequences (genes) by combining ESTs into groups where the members of the group show no sequence variation and thus represent the haplotype or partial haplotype (pHap) of each member of a gene family. Once gene sequences within a family were identified, examination of the structural differences between the members of the gene family was performed. Finally potential alleles for these genes are identified by comparing the ESTs from other genotypes to these sequences.

### Gene identification

We identified 45,255 pHaps in 12,702 contigs that were present in the cultivar Williams/Williams 82 EST libraries. In addition, approximately 35,000 Cap3 singletons appear to have coding potential. Since the majority of the EST sequences are assumed to represent random parts of genes, some of these singletons may in fact be partial reads of genes represented in the pHap collection which have no overlap with other pHaps from the same gene or they may represent single reads of rare messages. Thus it is safe to say that there are at least 45,000 soybean genes represented in the EST collection examined when both the pHap and singleton sequences are included.

### Sequence variation within gene families

Genes and paralogs of genes were most often differentiated by single base substitutions with inter-gene indels approximately 12 times less frequent (Table 2). This would be expected assuming a random mutational process was acting upon the gene sequences. However, approximately 10% of the base changes were found in runs of consecutive positions in the sequence (Fig 1A). Almost all of these consisted of two adjacent changes although 1% of the single base substitutions were in a run of three and a few were in runs of more than 10. This pattern of substitution between presumed paralogs could represent inter-codon dinucleotide preference which has been noted in plants by De Amicis and Marchetti [23]. These authors identified a bias in the combinations of nucleotides that separate adjacent codons. In their observations, the nucleotide in the 3^rd ^position of a preceding codon biased the use of nucleotides in the 1^st ^position of the following codon. Thus the presence of a large percentage of consecutive substitutions could be the result of the bias on acceptable codons imposed by the codon immediately upstream in a functional paralog.

Intriguingly, the most common indel between members of a gene family was a single base (Fig. 1B). Although this finding is not unprecedented [24,25], it was unexpected as a single or even paired one-base indels would be expected to disrupt the reading frame.  Examination of the sequence of the selected amplicons relative to the potential gene sequence (pHap) from which the primers where derived indicated that in both types of sequences (single/multiple pHap families) single base deletion/insertions were the predominant non-LDSDS sequence difference observed between the amplicon and the pHap sequences. These insertions/deletions were not associated with single base runs typical of polymerase "stuttering", but appear randomly distributed in the various sequences. In cases where forward and reverse reads of the PCR amplicons were obtained, these insertion/deletions were not confirmed indicating that, at least some of these discrepancies were the result of random base calling errors. Whether most of the apparent single base insertion/deletions observed in the EST sequences and as a consequence the pHap sequences represent random sequencing errors inherent to the single-pass sequencing of EST libraries or whether some are biologically relevant remains to be determined.

Insertion of long sequences consistent with introns also appear to be frequent in the gene structure of soybeans. In the 4.5 kb of coding sequence examined, 13 introns were identified indicating that introns are a common feature of gene structure in soybean. Based on these data, an intronic sequence will be encountered approximately every 350 bp of coding sequence with the size of the intron being ~220 bp.

### Paralog evolution in soybean

Since ESTminer families are sequenced based, they are assumed to represent only highly similar gene sequences such as those of the most recent gene duplications. Therefore, if all paralogs are retained after a large scale genomic duplication, there should be an excess of families consisting of 2 or more sequences. Allowing for random gene loss there should be an excess of gene families of 1–2 sequences. This has been observed in *Arabidopsis thaliana *[26] that has also undergone multiple rounds of genome duplication [27-30].

In the current study, most gene families were predicted to contain a single sequence (Table 1) based on representation in the EST libraries examined. However, PCR amplification and sequencing of amplicons from 8 of 9 single pHap families presented evidence of multiple related sequences in the genome of Williams 82. In all, 12 of the 13 (92%) loci examined, presented multiple product bands from PCR amplification or the presence of more than one sequence in the sequencing traces. These data are similar to the observations of Shoemaker *et al*. [4] which indicated that approximately 90% of the examined loci were present in multiple copies based on hybridization data. If these results are extrapolated to the genome as a whole, approximately 90 – 92% of all loci are present in multiple copies in this genome. Thus it is more accurate to say that the "single" pHap families contain few members where the "multiple" pHap familes contain many members.

On the basis of data derived from genomic sequence it appears that most genes are present in multiple copies, however transcriptional evidence in the form of representation in EST libraries indicates the opposite. This could be explained in a number of ways. First, the copies of the genes in question could be non-expressed (pseudogenes) and thus amplify by PCR as in this study and hybridize to molecular probes as in Shoemaker *et al*. [4], without producing a transcript. Gene duplication is thought to be followed by a relaxation in purifying selection on one of the copies[31]. In this system, the duplicated gene is freed to suffer random mutations without the counter force of negative selection. Since random mutations are considered to be mostly deleterious[32], it would be assumed that, in general, most random mutations would ultimately lead to the inactivation of the coding sequence by any number of means including mutations to their transcriptional promoters rendering them transcriptionally silent and thus not represented in the EST libraries.

Secondly, the EST libraries could represent a very shallow sampling of the transcriptome of soybeans. While formally a possibility, this dataset was composed of nearly 200,000 EST sequences which would make it the 11^th ^largest plant EST collection in dbEST at the time of this writing. In addition, 16 tissues subjected to various treatments [33] were sampled indicating that it is a broad sampling of the transcriptome in terms of tissues and developmental stages sampled. However, it must be noted here that the libraries from which the ESTs were derived were not normalized thus the libraries are expected to be a biased sampling of the transcriptome. In light of this, severely restricted expression of one of the still functional paralogs could also be a factor in the apparent excess of single gene families of pHaps in relation to the genomic data. Spatial and/or temporal restriction of expression of functional paralogs has been observed in a number of systems [34,35]. Additional studies provide evidence that subfunctionalization [36-38] maybe associated with duplicated genes in a wide array of eukaryotes including plants. An alternate fate of duplicated genes may be neofunctionalization[39] where one of the copies eventually acquires a new function altogether [40-42]. Either condition could lead to a sufficiently reduced expression pattern for one of the copies such that it was not captured in the un-normalized EST libraries but yet retains enough sequence similarity to cross hybridize to PCR primers or Southern blot probes.

Finally, the copies of duplicated genes (paralogs) could be so young or are under such selection that they still retain an identical sequence over most of their coding region. Thus, if a conserved fragment of two different genes were the only representatives of those genes in a gene family EST collection, then both of those genes would be represented by a single pHap sequence. While this my explain a small portion of the single sequence families, it probably can not be responsible for the majority of the cases given current models of duplicate gene evolution and the time scales predicted for the large scale duplication events identified by Shlueter *et al *[6].

### Functional classification of genes in gene families

While there are inherent difficulties associated with functional assignments using GO terms (Biased subset of terms, use of "Unknown" for functional annotations and a significant proportion of "no hits") it still may allow the observation of the larger trends in gene/paralog evolution. Another difficulty revolves around the definition of a "gene family" with different authors assigning family membership in various ways. For example, in *Arabidopsis*, the TAIR project recognizes 863 families [43] where Maere et al [44] recognize at least 3,472 families. For these and other reasons, we limited our conclusions to the more pronounced differences between the gene family groups without assigning absolute frequencies to the observations.

Family member designation in this study was based on sequence similarity and not necessarily by biochemical function, thus ESTminer families are undoubtedly discrete subdivisions of the biochemically and structurally defined gene families. This can be observed easily in the broad category of transferases seen in this study. While these genes all have one biochemical function (transferring a molecular moiety) they may actually transfer quite different molecules ie glutathione, hexoses, amino groups, etc. Because of this they have very different substrate binding domains. Thus, ESTminer recognizes the differences in sequence and separates them into smaller sub-families. This logic would extend to all of the broad categories represented in the GOslim terms. With this in mind, we look at the larger trends in the distribution of functional categories within the ESTminer gene families.

In general the distribution of functional classifications among the gene families identified in this study does not appear to be random. Examination of Figure 2 indicates that a number of functional categories were not randomly distributed amongst the gene families with 10 classifications departing significantly from expectations (asterisks). In almost all cases, single gene (pHap) families where the predominant class of gene family associated with each GO category. Only in the categories of structural molecule and RNA and lipid binding activity were multiple gene (pHap) families the largest class. This observation was unexpected since single gene (pHap) families were the largest class of gene family overall; comprising approximately 67% of all of the families. This may indicate that genes associated with structural, RNA and lipid binding proteins were either more apt to be duplicated, or that these genes once duplicated as a result of the two rounds of large scale duplication observed by Schlueter *et al *[6], were retained whereas the duplicated genes in other classifications were lost or diverged significantly in primary sequence. Similarly, in a study of *Arabidopsis *duplicated gene families using a modified GOslim annotation system, Maere et al [[44] supplemental data] observed that genes involved in structural activities were slightly more apt to be from gene families containing 3 or more members than those containing just 2 members indicating a trend for larger gene families as observed here. The trend was stronger in genes involved in lipid metabolism however about equal numbers of RNA binding genes were observed in each category (less than or equal to 2, 3 or more).

When individual gene sequences (pHaps) are considered, genes from multiple gene families are in the majority (Fig. 3) but the number of genes in the individual GO categories does not appear to be randomly distributed. A number of GO categories appear to contain multiple gene families that have more than the average number of members (Fig. 3 red asterisks). Theses categories (receptor, protein and lipid binding and catalytic and enzyme regulator activities) have proteins whose functions likely involve specific structures with particular residues contributing to their functional activities, thus a small number of minor sequence changes can change their substrate or binding properties drastically.

On the other hand, some GO categories appear to contain families with a smaller than expected membership (Fig 3, blue asterisks). These activities (hydrolyase, transcription factor, RNA binding, kinase, oxygen binding, transcription regulator, signal transducer, nuclease and carbohydrate binding) likely involve proteins with very different three dimensional structures yet they perform common functions within each group. As a consequence, their primary nucleic acid sequences are distinct, thus they form smaller ESTminer gene families.

In an effort to determine if specific functional terms were over-represented in families with few pHap members, analysis of the distribution of specific terms was performed using the BLAST2GO program [22]. This program performs a multiple comparison of all GO terms in a reference set of gene annotations against a subset of those annotations and indicates which GO terms are over-represented in the subset using FDR correction. This analysis indicated that 50 GO functional terms were over-represented in this dataset compared to all of the gene families (many + few pHap families) again indicating that the functional classes were not randomly distributed in the two groups. The largest classes of over-represented terms included genes whose functional annotations were involved in transferase (36%), porter/transporter/permease (18%), kinase (10%), binding (10%) and hydrolase (4%) activities. In a study of duplicated genes in *Arabidopsis*, these categories also tended to contain families with relatively few members[44]. These observations may indicate that these functional categories are composed of genes with more discrete physical structures or functions. In a different study of duplicated genes in *Arabidopsis*, Blank and Wolfe [35] also observed that duplicated genes with functional annotations involved in kinase (14%) and transporter (19%) functions were more apt to remain duplicated than other functional categories during *Arabidopsis *evolution. Because most "single" gene families in this study actually contain 2 or more sequences, it is possible that these data are also consistent with that seen by Blanc and Wolfe since kinase and transporter functions were associated with gene families with few members (single) in this study. If so, these data also suggest that the categories of genes which remain duplicated in different taxa following large scale genome duplication events could be subject to comparable selective pressures in each taxa reflecting a similarity in environmental forces applied to each species. This may become apparent when sympatric and allopatric congeneric species comparisons are performed.

## Conclusion

We have developed ESTminer [18], a suite of Perl scripts that use Cap3 and BLAST to cluster and align related ESTs and then do an exhaustive analysis to identify every unique sequence variant in the cluster. The procedure was applied to the ESTs from the homozygous soybean cultivar Williams/Williams82 and resulted in the identification of 12,702 presumed gene families with an average of 3.6 paralogs/family. Analysis of the differences which define pHaps (genes) within a gene family indicate that single substitutions account for most of the substitution variation observed (90%). The next most frequent substitution event observed was 2 consecutive base substitutions. This observation could be the result of codon bias associated with non-independence of codon choice imposed by the preceding codon[23]. Introns appear to be frequently encountered in the coding region of soybean genes. We observed frequent insertions consistent with introns in the 4.5 kb of genomic sequence examined. Extrapolated to the transcriptome of soybean, an intron of approximately 220 bp will be encountered every 350 bp of coding sequence.

A majority of all gene families represented in the EST libraries contained a single gene where genomic amplifications indicated the presence of more than 2 genes for most of the loci examined here. This suggests that many of the duplicated genes in this genome are either transcriptionaliy silent or have a restricted expression such that they were not captured in the EST libraries or if captured in the libraries, they exist in few copies and were removed from consideration due to the validation procedure. However, under the classic model of duplicate gene evolution, most duplicated genes would be expected to quickly become transcriptionally/functionally inactivated due to the effects of deleterious random mutation. Thus the finding that there are two or more genomic loci for each single member gene family sequence could also be explained under this model of duplicated gene evolution.

The majority of these results are from *in silico *analyses. However, these data allowed the examination of 13 gene families. In multiple gene families (4) all sequences derived from genomic DNA were consistent with one or more pHaps in the family in overall sequence similarity and the presence of 96% of the ESTminer predicted LDSDs indicates their reliability as gene defining differences. Therefore, these pHaps represent a significant step forward in the molecular biology of soybean. These results will be used to develop useful genetic markers and apply them to soybean breeding as well as in broader studies of plant molecular biology and genome evolution. Further, our procedure could be used in other plant or fungal species where highly inbred or isogenic lines are available or in haploid bacteria. In cases where inbred lines are not available, ESTminer is still applicable because the LDSDs identified will be valid. The only ramification is that the number of pHaps will be increased by the number of alleles of each gene and thus the total pHap count will be a poorer estimator of the number of genes represented in an EST library. Thus, EST analysis using ESTminer would be a significant contribution to research in those plant species that have few genomic sequences available and no current genome sequencing project planned.

## Methods

To identify genes and alleles we used the ESTminer suite of PERL programs [18]. The functions of these programs are briefly described below.

### EST collection

Approximately 300 k EST sequences for *G. max *from the Public Soybean EST project [2] were obtained from dbEST [47] on 01-02-2003. This collection was processed to remove those sequences less than 100 bp in length and to delete adinosine- and thymidine-rich regions from the 5' or 3' ends of the remaining sequences. The sequences were subdivided into groups based on the source's genotype: All-Williams (AW) derived from the closely related and highly inbred cultivars Williams and Williams82 [44,45] and Non-Williams (NW) derived from all other *G. max *cultivars.

### Gene family consensus sequence assembly

Gene family consensus sequences were assembled from 196,867 AW and 93,653 NW ESTs using Cap3 [19] with default assembly parameters except for the Cap3 parameters of the minimum EST overlap of 21 bases (-o 21) and sequence trimming of the first and last 10 bases (-y 10).

### Gene family EST collection

A database containing both the AW and NW ESTs along with the Cap3 consensus sequences (included to facilitate subsequent analyses of the alignments) was assembled. BLASTN was used to assemble putative gene family-specific alignments using the Cap3 consensus sequences as the queries with an expectation value of 1e-9. Low complexity filtering was turned off in order to align the entire EST sequence even in low complexity regions of the EST's sequence. This reassembly step was required to ensure that all relevant ESTs were included as our experience with Cap3 and the large soybean EST collection showed that the Cap3 assemblies were often incomplete. Non-Williams ESTs were included to allow the identification of allelic differences between the various genotypes.

We observed that ESTs could be contained in a BLAST alignment due to a relatively short shared motif. To remedy this, we removed any EST that had less than 90% of it's sequence included in the BLAST alignment. Next, EST clusters representing individual pHap were identified by dividing the AW ESTs that remained in a BLAST alignment into classes where each contained ESTs with no sequence differences in their overlapping regions. Unfortunately, this method of identifying genes is complicated by two related facts. First, the EST collection is a snapshot of the transcripts at a particular time. If the members of a gene family are not transcribed at identical rates in that tissue, the relative abundance of ESTs in the library will reflect this and a poorly expressed gene may be represented by a single EST. Second, ESTs are single-pass DNA sequences and can be expected to contain errors. Thus a unique EST due to a relatively low level of expression is indistinguishable from one that is caused by a sequencing error. To reduce the times that we identified a sequencing error as a transcript from a poorly expressed gene, we required that any sequence difference be seen at least twice in the entire soybean EST collection, thus removing both sequences with random errors and truly unique transcripts from further analysis.

### Gene identification

After BLAST analysis and the quality control steps described above, each BLAST alignment represents a collection of EST sequences from the highly related genes in a gene family. Each gene family, thus defined, is composed of one or more clusters of ESTs with the members of each cluster showing no internal sequence variation. When there was a single cluster representing a single gene family, the constituent ESTs were combined to create a single sequence, which was defined as the potential gene sequence (pHap) for that family. In cases where there were two or more such clusters in the gene family, the sequence differences between them were termed locus defining polymorphisms (LDSDs). Each individual EST cluster in the gene family was collapsed to a single representative of the cluster's unique sequence in a two step process as previously described [18]. Thus, the some times large collection of redundant EST sequences in a gene family were reduced to single representatives of each potential gene sequence (pHap) from that family based on their shared sequence differences. These shared characters represent locus defining sequence differences (LDSDs) or locus defining polymorphisms (LDPs) that define individual genes (pHaps) in a gene family.

### LDSD validation

To attempt to validate the existence of the predicted locus defining sequence differences (LDSDs) in selected gene families, PCR and sequencing primers were designed by hand from pHap sequences. The 18 bp primers were designed to contain ~50% GC were possible. Optimum annealing temperatures were empirically determined for each primer. The standard PCR reaction contained 2.5 μM primers, 1 × Epicenter Master Amp PCR Premix B, 0.06 U Invitrogen Taq DNA polymerase and 50 ng genomic DNA. A "touchdown" cycling parameter was used (94 C 45 sec. elongation 70 C 30 sec. (-1 C each cycle) 72 C 45 sec.) × 4, (94 C 45 sec., 65 C 30 sec., 72 C 45 sec) × 29, final 72 C extension 2 minutes. Primers internal to the PCR product were used as sequencing primers.

PCR products were separated on 1% Invitrogen L.M.P. agarose in 1 × TAE buffer. The majority product band was excised from the gel and released from the matrix with 1 U/200 mg gel Epicenter GELase Enyzme according to the manufacturers recommendations. DNA was precipitated with ethanol. The pellet was resuspended in sterile water and an internal sequencing primer was used for sequencing.

DNA sequencing was performed at the ISU DNA Sequencing and Synthesis Facility using BigDye 3.1 (Applied Biosystems) chemistry and run on an ABI 377 and 3100 DNA sequencers using base caller versions 3.3.1b2 and BC 1.5.0.0 respectively. Sequence traces were manually scored and compared to pHap sequences.

### Functional annotation of gene families and pHaps

Functional annotations were assigned to gene family consensus sequences and potential gene sequences (pHaps) by BLASTX analysis using a SeqDBlite database[21]. This database contained approximately 85 K highly annotated protein sequences from a variety of organisms and is internally redundant with products from multiple species annotated with the same GO term. BLASTX analysis was performed using an expectation cut-off of 1 × 10^-4^, filtering ON. The functional annotation inferred for each pHap was taken from the best BLAST hit. Since not all high hits contained a functional annotation and a query sequence could be similar to gene products from multiple species each having the same annotation, the GO identification numbers for the 10 highest hits were collected. These identifiers where then matched to their respective plant GOslim term and a non-redundant list of GOslim terms for each sequence was collected using Perl scripts. In this manner, the majority, but not all, of all sequences resolved to a single plant GOslim functional term. For BLAST2GO analysis, the BLASTX analysis was performed locally on the single member gene families and the GO terms of each hit were extracted by Perl programs. These files were then used as input into the Blast2GO program. The significance level employed in all statistical calculations was p = 0.05. BLAST2GO also allows the selection of a significance level for the False Discovery Rate (FDR) which was used as a cut-off at a 0.05% probability level.

### Hypothesis testing

Hypothesis testing was performed using the Chi-squared statistic with 1 df. A goodness-of-fit test was performed on the distribution of GOslim terms within gene families (Fig. 2) assuming that 67% of all families contained few members (single sequence families). For the distribution of GOslim terms within all of the potential gene sequences (pHaps) (Fig. 3) a goodness-of-fit test was performed on the distribution of individual pHaps in each GOslim category using the assumption that the average multi-gene family contained 9 members (number of multi-gene pHaps/number of multi-gene families). The number of gene families associated with each Goslim category was taken from Table 3 and multiplied by 9 to arrive at the expected value. The significance level chosen was p ≤ 0.05 in both cases.

### Sequence availability

pHap, singleton, and family consensus sequences, original BLAST alignment and a searchable database of the pHap sequences is available at the SoyBase web site [48].

## Authors' contributions

RTN wrote the software, performed the *in silico *analyses and prepared the manuscript. RCS conceived and supervised the study. All authors have read and approved the final manuscript.
